# Silent cells? Potential for context-dependent gene expression in mature sperm

**DOI:** 10.1098/rspb.2024.1516

**Published:** 2025-01-08

**Authors:** Rowan A. Lymbery, Francisco Garcia-Gonzalez, Jonathan P. Evans

**Affiliations:** ^1^ Centre for Evolutionary Biology, School of Biological Sciences, University of Western Australia, Crawley, Australia; ^2^ Department of Biodiversity, Conservation and Attractions, Kensington, Australia; ^3^ Doñana Biological Station (EBD-CSIC), Isla de la Cartuja, Sevilla, Spain

**Keywords:** haploid expression, fertility, ejaculate-mediated paternal effects, epigenetic inheritance, sperm competition, sexual conflict

## Abstract

Sperm are traditionally viewed as transcriptionally and translationally silent cells. However, observations that components of the cellular machinery of gene expression are maintained in ejaculated sperm are increasingly cited as challenges to this fundamental assumption. Here, we critically evaluate these arguments and present three lines of evidence from both model and non-model systems that collectively raise the question of whether ejaculated sperm may be capable of active gene expression. First, and critical for arguments surrounding the possibility of differential gene expression, we review recent evidence that spermatozoa may retain the capacity to transcribe and translate their genomes. Second, we highlight how sperm cells can exhibit differential transcript quantities across different post-ejaculation environments. Third, we ask whether the accumulating evidence of remarkable phenotypic plasticity in post-ejaculatory sperm phenotypes could be mechanistically underpinned by changes in sperm gene expression. While these lines of evidence are indirect and do not definitively show transcription of sperm genomes, we highlight how emerging technologies may enable us to test this hypothesis explicitly. Our review advocates for progress in this field and highlights several important evolutionary, ecological and practical implications that will probably transcend disciplines to the clinical and applied reproductive sectors.

## 1. Introduction

Historically, sperm have been viewed as transcriptionally and translationally silent cells with phenotypes determined by the male during spermatogenesis, in essence reducing their function to simple ‘DNA-delivery machines’. This paradigm is based on two key observations of sperm development, typically in humans and mammalian model systems (see box 1). First, during spermatogenesis in these model systems, DNA becomes densely packaged and the majority of histones (binding proteins that help support the DNA) become replaced by protamines, which cause an increase in the folding of DNA and make it inaccessible for transcription [2]. Second, during sperm maturation, the cytoplasm is substantially reduced, leaving little space for transcriptional and translational machinery [1]. However, as our knowledge of sperm biology rapidly expands across different taxa, exciting developments have led to some researchers questioning this longstanding dogma of ‘silent sperm’ [9,10].

Box 1: **The ‘dogma’ of silent sperm**

In the fields of genetics, cell biology and molecular andrology, most researchers endorse the view that during spermatogenesis, the transcriptional activity of developing spermatids becomes diminished. According to this view, mature spermatozoa are optimized for the transport of the paternal genome to the egg—nothing else [1].

One of the key stages of spermatogenesis in mammals and many other taxa involves the compaction process, which occurs when histones (the proteins around which DNA are wrapped) are replaced by protamines, which are smaller proteins that facilitate tighter compaction of the sperm and are assumed to render the sperm’s DNA inaccessible to transcription machinery (transcription factors, RNA polymerase etc.) [2]. Furthermore, in the final stages of spermiogenesis, maturing spermatids undergo profound architectural changes, which result in the loss of most of the cell’s cytoplasm, which along with the translational machinery is absorbed by Sertoli cells [1]. This process of cytoplasmic reduction ensures that mature spermatozoa are highly compact, motile and agile cells able to locate and penetrate the egg. Accordingly, transcriptional and translational machinery is assumed to be absent or significantly diminished in mature sperm cells, which is supported by studies that have tested but failed to detect nuclear transcription in late spermatids and mature spermatozoa (reviewed in [3]; but see below) and those reporting that long-lived messenger RNA (mRNA) from diploid cells can be translated in mature spermatozoa [4].

Despite the expectation that mature spermatozoa are transcriptionally and translationally silent cells, sperm carry thousands of RNAs, including mRNA, microRNAs (miRNA), interference RNAs (iRNA), and so on (e.g. [5]). Until relatively recently, these large quantities of RNAs in mature sperm were thought to exist merely as relics of spermatogenesis [6,7]. However, as we describe in this review, the extent of compaction of sperm DNA is not absolute and differs taxonomically, leaving some authors to question whether the presence of sperm RNAs may allow for the timely expression of certain portions of the sperm’s haploid genome (e.g. [3]). Indeed, in an early rare experimental test for transcription by mature human sperm, Naz [8] reported that the addition of transcription inhibitors altered the number of acrosome-reacted sperm. Despite this tentative evidence, as we outline in this review, very few other published studies have directly tested whether mature, ejaculated sperm transcribe new RNAs.

We now know that mature, ejaculated sperm in many species retain many more of the cellular elements of gene expression than just DNA. For example, studies have found that sperm contain complex populations of messenger and non-coding RNAs, which have been linked to fertility and fertilization, suggesting that they may contribute to sperm function and fitness [10–12]. Meanwhile, evidence has accumulated suggesting that these RNAs originate from post-meiotic transcription in developing haploid sperm, rather than being simply ‘loaded’ into the sperm by diploid male cells (box 2). However, it is still widely accepted that transcription of nuclear DNA ceases in mature, ejaculated sperm.

Box 2: **Haploid gene expression in developing spermatids**

It was long assumed that transcription in gametic cells of male animals ceased by the time of the first meiotic division, i.e. before the transition from diploid to haploid genomes. However, in the last decade, several studies have provided unambiguous evidence for de novo postmeiotic transcription of numerous genes in developing spermatids, the haploid male germ cells generated after the first meiotic division of spermatogenesis (e.g. [13–15]; reviewed in [9,16]). Nevertheless, the spatial organization of spermatogenesis, where haploid spermatid cells are connected to each other via cytoplasmic bridges [17], cast doubt on whether this postmeiotic transcription represented ‘haploid expression’ in the sense of individual sperm cells expressing their own genes, or whether sperm are effectively diploid owing to transcript sharing across bridges. Only very recently has evidence arisen that developing sperm retain substantial numbers of transcripts from their own haploid genome. For example, Bhutani *et al*. [18] found allelic expression biases (i.e. overexpression of transcript products from a particular haploid allele at a locus) that correlated with the genotypes of haploid spermatids for numerous genes in mice, humans, primates and bulls. Moreover, they found putative evidence that these gene products were localized in subcellular regions that prevented their transport across cytoplasmic bridges [18]. Thus, it appears that in many species true haploid expression is likely in postmeiotic spermatids, and sperm phenotypes may often reflect their haploid genomes (see also [19–21]). What remains controversial is whether transcription of genes occurs after spermatogenesis in mature ejaculated sperm; a question that forms the basis of this review.

**Figure 1.**
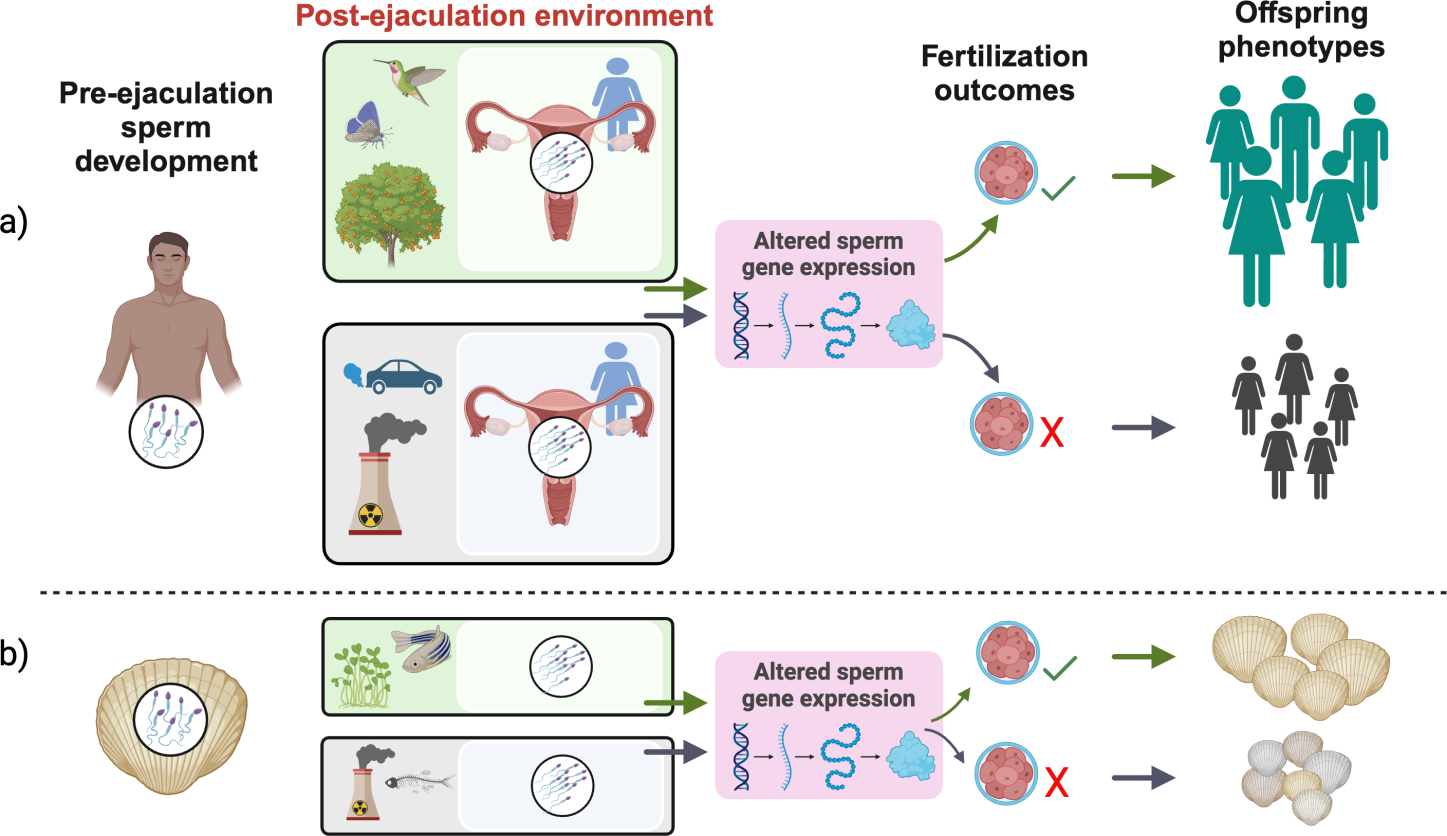
Schematic overview illustrating context-dependent gene expression in mature, ejaculated sperm and some of its potential implications. Sperm from a single individual typically experience a range of post-ejaculation environments in both internally (*a*) and externally (*b*) fertilizing species. These different environments (female phenotypes, female environments (e.g. female reproductive tract environments or ovarian fluid traits), exposure to chemicals, social environments, stress owing to environmental variables such as temperature, etc.) may elicit sperm phenotypic plasticity and potential differential gene expression. This context-dependent gene expression has important implications for sperm function, fertilization outcomes, and, owing to ejaculate-mediated paternal effects (see [22]), for offspring phenotypes (e.g. development, viability, behaviour and behavioural disorders, metabolism, health, longevity) and even, potentially, the phenotypes of ensuing generations. Here, different offspring sizes represent differences in hypothetical fitness/quality. Created in Biorender [23].

This review is structured into three sections that summarize indirect, but suggestive, evidence that mature, ejaculated sperm might be capable of actively transcribing and translating their nuclear genes. First, we highlight how, in some species, mature ejaculated spermatozoa retain important components of the cellular machinery that are required for transcription and translation of nuclear DNA. Second, we review studies reporting that RNA transcript quantities in mature sperm can depend on the environmental context (see figure 1), which could be interpreted in some cases as active transcription of sperm genes. Third, we ask whether sperm gene expression may, under some conditions, underlie patterns of phenotypic plasticity by individual sperm within an ejaculate. Importantly, we stress that none of these findings provides definitive evidence for sperm nuclear gene expression; indeed, we argue that direct tests of this possibility are badly needed. Accordingly, our review concludes by suggesting key priority areas for future research, including the use of emerging technologies to label and visualize nascent (newly synthesized) RNAs in mature spermatozoa and highlighting the experimental use of non-model systems to test these ideas explicitly. We also explore some of the unique biological and practical implications of gene expression by mature sperm in response to changes in the post-ejaculation environment (see figure 1), which extend from the clinical fields of assisted reproduction to the fundamental fields of natural selection, sexual selection, paternal effects and sexual conflict.

### (a) Preamble

Before we commence our review, several key points are worth emphasizing. First, we focus on the potential for ‘ejaculated sperm gene expression’, which would entail the mature, ejaculated spermatozoan’s genome synthesizing RNAs and proteins that ultimately affect the sperm’s phenotype. We acknowledge that once sperm cells are fully mature, they are typically stored in the male reproductive tract for a period before ejaculation and that mechanistically, both stored sperm and ejaculated sperm might be expected to have the same cellular capabilities (including gene expression or lack thereof). However, many of the studies we discuss in this review focus on ejaculated sperm, as it is more tractable to experimentally expose ejaculated sperm to the environmental variation that might trigger sperm gene expression, while controlling for potential confounding mechanisms. For example, changes in transcript profiles of sperm stored in the male reproductive tract may represent uptake of RNA from diploid gonad cells or from seminal fluid ([24]; see box 1).

Second, we consider ejaculated sperm gene expression to be a specific form of ‘haploid gene expression’, a term that might otherwise refer more broadly to the less controversial concept of expression by the haploid genome at any point after the first meiotic division (e.g. in immature, developing sperm; see box 2).

Third, we focus on expression of nuclear genes rather than mitochondrial genes, unless we explicitly state otherwise, as it is the nuclear DNA that is expected to be inaccessible to the transcription ‘machinery’ in mature sperm (box 1).

Fourth, while our article will include much discussion of sperm RNAs, our purpose is not to review their functions (known or speculated; interested readers can refer to previous reviews on this topic [10–12]), but rather to explore their relevance as indicators of active gene expression by ejaculated sperm. However, as discussed throughout our review, the existing evidence makes it difficult to draw categorical conclusions as to whether RNAs are transcribed by the sperm or taken up from diploid cells or seminal fluid. Disentangling these pathways offers promising new insights (see below).

Finally, our article focuses on the potential for translation and, critically, transcription of mature, differentiated DNA in animal sperm, rather than in plants, where sperm and pollen cells are well known to be transcriptionally active [25]. However, many of the evolutionary implications of ejaculated sperm gene expression that we propose are novel and relevant to both animal and plant systems.

## 2. Transcriptional and translational machinery in sperm

As we note above, the broadly accepted view of spermatogenesis, based on mammalian model systems, is that by the time of sperm maturation the sperm genome has become condensed and inaccessible to transcription, and that the reduced cytoplasm has lost the ribosomal machinery necessary for protein translation [1]. However, there is now good evidence that de novo protein translation can occur in ejaculated sperm [26–28], and suggestions that transcription might be possible in mature and ejaculated sperm in some systems [28,29].

In a landmark study, Gur & Breitbart [26] incubated ejaculated human sperm with labelled amino acids and found that these were incorporated into newly translated, nuclear-encoded proteins during capacitation. Intriguingly, this process was blocked by inhibitors of mitochondrial translation complexes, but not by inhibitors of cytoplasmic ribosomes (where translation of nuclear genes occurs in most cells). Furthermore, inhibition of mitochondrial translation adversely affected sperm motility, capacitation and *in vitro* fertilization success (see also [30]). Similar results were obtained for nuclear-encoded proteins in ejaculated mouse sperm [27]. Interestingly, recent work in humans has also shown that when mitochondrial DNA is eliminated during spermatogenesis, mitochondrial transcription factor is re-located to the sperm nucleus [31]. Thus, despite the reduction of standard cytoplasmic ribosomes, it appears that nuclear-encoded proteins can continue to be translated via mitochondrial-type gene expression machinery in mature sperm. However, despite these key findings, the potential roles of translation in sperm function and fertility have largely been ignored until recently.

In contrast to the traditional assumption that sperm have lost translational machinery, recent studies within the assisted reproduction literature report that ejaculated sperm contain abundant transcripts that code for ribosomal proteins (e.g. in bull sperm [32]), that important chaperone proteins with multiple cellular functions increase in expression during post-ejaculation storage and capacitation (e.g. HSP90 in human sperm [33]) and that translation inhibitors suppress protein synthesis, mitochondrial activity and ultimately sperm swimming velocity (e.g. in boar sperm [28]). Moreover, although the study of sperm protein translation outside of model species has largely been neglected, there is some evidence in external fertilizers that inhibition of mitochondrial translation during late sperm maturation prevents sperm from acquiring full motility upon spawning (e.g. in seabream and zebrafish [29]). Therefore, it is possible that mitochondrial protein translation of nuclear-encoded genes is conserved and functionally important for ejaculated sperm, at least in some species. Furthermore, cytoplasmic ribosome complexes may be functionally retained in sperm of some taxa. For example, recent studies in *Drosophila melanogaster* indicate that sperm of fruit flies retain almost the full suite of ribosomal proteins and, intriguingly, patterns of paralogue specialization and switching in these proteins compared to other testes tissue, suggesting functional specialization of ribosomes for sperm [34,35].

The assumption that sperm DNA is inaccessible to transcription owing to extreme condensation is not universal across animals; while many taxa do use protamines or similar nuclear basic proteins to package sperm DNA, some invertebrate groups, including sponges, comb jellies and crustaceans, lack such proteins entirely and retain normal histone arrangements in mature sperm [36]. Interestingly, recent work in flowering plants (where sperm also lack protamines and gene expression is widely accepted) shows that sperm chromatin condensation in these systems can be achieved without compromising transcription [37]. Furthermore, even in model systems with highly compact sperm genomes such as humans and mice, histones are retained at specific and consistent DNA sequences, potentially allowing transcription of these genes in mature sperm or early embryos (e.g. [38–40]). An interesting avenue for future research would be to compare patterns of histone modifications or other epigenetic marks (e.g. DNA methylation) between stored and ejaculated sperm, which might reflect changes in the genes that are open to transcription. There is also preliminary evidence from externally fertilizing fishes that transcription of genes in mature sperm during storage in the male reproductive tract may be required for post-ejaculation motility [29]. It is important to note that, unlike the evidence for translation of nuclear genes, none of these findings provides definitive evidence for nuclear transcription in ejaculated sperm. Nevertheless, coupled with findings of up-regulated RNA expression in sperm in response to post-ejaculation environmental changes in a range of species (see below), these results lead us to speculate that both transcription and translation of genes in ejaculated sperm might be required for full sperm functionality, at least in some systems.

## 3. Differential RNA abundance in ejaculates

Many protocols employed in assisted reproduction studies offer good opportunities to test the environmental dependency of RNA quantities in mature sperm. For instance, comparison of fresh versus frozen-thawed sperm in a variety of species, including humans, has shown that cryopreservation leads to altered RNA quantities [3,41,42]. As an example, Dai *et al*. [42] found 567 messenger RNAs (mRNAs) and 135 microRNAs (miRNAs) that were differentially abundant in frozen-thawed boar sperm when compared to fresh samples; notably, some of these changes were owing to up-regulation of transcripts (see [43,44] for similar findings on humans and pandas, respectively). Down-regulation of RNA could be explained by sperm cryoinjury, perhaps inducing inefficient protein translation or higher susceptibility of mRNA to degradation [3]. While the up-regulation of sperm transcripts after cryopreservation is possibly consistent with active transcription, it is important to note that relative up-regulation could also be observed if RNA molecules exhibit differential stability with regard to cryopreservation (with standardization of expression data leading to the appearance of ‘up-regulation’ for more stable transcripts; e.g. [45]). Therefore, it is possible that putative transcriptional differences could be a statistical artefact, and we advocate for direct tests of de novo sperm transcription in a range of systems (see §5).

Changes in mRNA abundances and protein levels after the sperm undergoes capacitation (the biochemical and physiological changes that mammalian spermatozoa undergo in the female reproductive tract and that are required to make sperm capable of fertilizing oocytes outside the male reproductive tract) provide further hints about the potential for active expression by ejaculated sperm DNA. Studies looking at these changes have experimentally separated sperm from seminal fluid (to control for potential confounding effects of RNA transfer into sperm cells), then have induced capacitation and measured sperm RNA abundance changes. They have documented, for the most part, mRNA down-regulation [33,46] but there are also reports of up-regulation of miRNAs and mRNAs during/after capacitation [47], suggesting that transcription of some genes may prepare sperm for fertilization. However, as discussed above, more direct tests are still required to determine whether ‘up-regulation’ truly represents de novo transcription. Remarkably, there are studies indicating de novo sperm translation during capacitation ([26,27,30,33]; see above). Thus, the protein synthesis component of gene expression can occur in ejaculated sperm within the female reproductive tract.

While most of the evidence for differential RNA quantities in sperm is confined to internal fertilizers, systems with external fertilization have the potential to provide unparalleled power to evaluate the context-dependency of gene expression in ejaculated sperm. Recent work on the broadcast spawning mussel *Mytilus galloprovincialis*, for example, exposed replicated samples of ejaculated sperm to two different temperatures (ambient versus heat stress) and reported the down-regulation of mRNA for heat shock products under stressful temperatures [48]. Whether these changes were owing to RNA degradation or transcriptional modifications was not established; indeed, either degradation or co-translational loss of transcripts may be the most likely explanations in this case. However, the authors argued that we might expect sperm of external fertilizers to exhibit considerable plasticity in both gene expression and phenotype, given they have no homeostatic protection from the unpredictable external environment once released [48]. Interestingly, preliminary findings from externally fertilizing fishes suggest that transcriptional capacity may be retained in mature sperm pre-ejaculation ([29]; see above). If confirmed, this could indicate at least the potential for ejaculated sperm from externally fertilizing species to rapidly adjust gene expression prior to fertilization. Further study of environment-dependent RNA expression in external fertilizers' gametes is needed to test these hypotheses.

## 4. Could sperm gene expression underlie phenotypic plasticity?

In both internally and externally fertilizing species, mature spermatozoa typically undergo post-ejaculatory phenotypic modifications owing to changes in the pre-fertilization environment [49], sometimes driven by females [50–54] or through ejaculate–ejaculate interactions [55–57]. Such factors can differentially regulate a diverse range of functionally important ejaculate phenotypes that influence fertilization, such as sperm motility and capacitation [49]. For example, studies in externally fertilizing fishes and marine invertebrates have used split-ejaculate designs to reveal that sperm motility, physiology and sperm chemotaxis within an individual male’s ejaculate can depend on the identity of the specific female (sperm chemoattractant) ‘donors’ that are crossed with a given male’s sperm [53,58,59]. Furthermore, the phenotypic changes in sperm within a male’s ejaculate that are induced by the reproductive fluid of specific females have implications for competitive fertilization success [60] and ensuing patterns of embryonic survival [59]. At the mechanistic level, such male–female interactive effects may depend on sperm detecting and responding to a complex and variable cocktail of ions and proteins that differentiate the reproductive fluids of different conspecific females [52,61]. Additionally, studies have used split-ejaculate or repeated measures designs in externally fertilizing fishes to expose sperm subsamples within a male’s ejaculate to different abiotic treatments (e.g. different temperatures and salinities) and found that sperm behaviour and swimming speed changes depending on the environment [62,63]. Thus, at least in species where split-ejaculate designs can be implemented, it appears that sperm may exhibit phenotypically plastic responses to a range of environmental cues.

At the molecular level, a common parsimonious explanation for phenotypic plasticity for many cell types is differential gene expression triggered by variation in environmental cues [64,65]. Thus, to the extent that the effects described above represent phenotypic changes of individual sperm cells, this accumulating evidence could present a challenge to the idea that mature sperm are transcriptionally and translationally silent cells. However, while protein translation may well occur in sperm, transcription remains unproven (see above), and there could be several alternative explanations for phenotypic plasticity in sperm cells that do not invoke transcription. For example, receptor-mediated internalization of external molecules, or differential modification or translation of proteins (without active transcription), might explain some post-ejaculation changes (e.g. [66]). Moreover, most studies measure average sperm phenotypes at the level of ejaculates, as it is logistically challenging to track changes in individual sperm cells in the majority of species. Therefore, another potential explanation for some environmental effects on ejaculate traits is that different environments select for different sub-populations of sperm within an ejaculate, which already express different phenotypes (e.g. [19]). Nevertheless, individual sperm do undergo behavioural and physical modifications when encountering female-derived factors [49,67], and some of the differential effects of females described above can unambiguously be ascribed to physiological changes in individual sperm cells [53,68].

If expression changes in the sperm haploid genome do underlie phenotypic plasticity across environments, we might also expect to see variation in the shape and magnitude of plastic responses (i.e. the ‘reaction norm’ across environments) among haploid sperm within an ejaculate. This prediction would depend on the genes that underlie plastic responses; if the diploid male was heterozygous (carried two different allelic copies of the same gene) for the hypothetical genes whose expression responded to environments, then haploid sperm carrying different alleles would be expected to have different phenotypic reaction norms. However, if the male was homozygous at the relevant genes, then all sperm within the male’s ejaculate would have the same phenotypic response to environments; i.e. identical reaction norms. Nevertheless, the existence of different phenotypic reaction norms among sperm within an ejaculate would constitute convincing (albeit indirect) support that the haploid sperm genomes were involved in phenotypic plasticity. This has been functionally challenging, if not impossible, to test in many systems, although species that produce genetically identical haploid sperm within an ejaculate may offer an experimental solution (see [69]). Furthermore, recent technological advances may offer opportunities to characterize reaction norms of individual sperm in other systems. For example, techniques to ‘tether’ sperm by their heads and record images/videos of individual sperm cells [70,71] could potentially be combined with microfluidic slides that allow different solutions to be pumped over sperm [72] to characterize behavioural or physiological changes of individual sperm as they encounter different environmental media. Such studies, and other methods of elucidating the mechanisms that underlie post-ejaculation phenotypic plasticity, should be an important priority for future research into the possibility of sperm gene expression.

## 5. Key priority for future research: could mature, ejaculated sperm transcribe nuclear DNA?

This brief synthesis of evidence highlights the need for direct tests of active transcription of the nuclear genome by mature, ejaculated sperm. As we highlight above, while it has become clear that ejaculated sperm can translate proteins, this does not necessarily imply transcription of nascent (newly synthesized) RNAs. Findings of post-ejaculation phenotypic plasticity and differential RNA expression could also be explained without invoking active transcription. However, in the light of evidence outlined in this review, and the rise of new powerful technologies (see below), we argue that it is timely for direct tests of post-ejaculation transcription. Indeed, post-ejaculation translation was once thought as unlikely as transcription, until direct tests revealed otherwise [26]. In particular, extending such investigations to non-model systems, such as external fertilizers, may be insightful, given that in such species sperm DNA packaging could be less restrictive [36] and the need to respond flexibly to post-ejaculation environments may be heightened.

A promising approach for testing the hypothesis of active post-ejaculation transcription would be to label and visualize nascent RNAs. Such a technique was employed by Vibranovski *et al*. [13] to provide direct evidence of post-meiotic transcription during spermatogenesis and could be extended to mature ejaculates. Moreover, new sequencing techniques that allow nascent RNAs to be captured and sequenced [73] offer the potential to identify any genes that are actively transcribed. Indeed, a recently published conference abstract reported active transcription of nascent RNAs from two genes in human sperm following exposure to follicular fluid [74]. An alternative (or complementary) approach would be to employ transcription inhibitors (e.g. actinomycin D) when testing for differential RNA expression or phenotypic plasticity of sperm cells under different environments. To our knowledge, only one published study has applied this approach to ejaculated sperm, with Naz [8] reporting that treatment with actinomycin D altered the acrosome reaction and fertilization capacity in human sperm. The opportunities to apply combinations of the above techniques to provide improved mechanistic understanding of these processes offer exciting avenues for further research.

## 6. Evolutionary implications of post-ejaculation sperm gene expression

The capacity for gene expression in mature, ejaculated sperm would have many important evolutionary implications. To be fully realized, some putative implications may require both transcription and translation of genes after ejaculation, but others would not. For example, the presence of post-meiotic transcripts from the haploid sperm genome, and differential translation of proteins encoded by the haploid genome (phenomena that have now been shown to occur in several systems) would be sufficient to generate many of the processes we discuss below.

### (a) Natural selection

As we highlight above, the potential for widespread phenotypic plasticity in sperm cells may enable them to respond to, and function in, variable post-ejaculation environments, although the extent to which such plasticity is adaptive will depend on whether it enables a specific genotype to endure greater environmental heterogeneity [75]. However, the possible evolutionary ramifications of such phenotypic plasticity have rarely been explored. Importantly, if post-ejaculation plasticity does indeed represent expression of the sperm’s genome, this could lead to greater within-ejaculate variation in sperm phenotypes as they respond to environments, owing to among-sperm variation in haploid genes coding for plastic traits and thus different phenotypic responses among sperm to the same environments. This in turn may increase the opportunity for haploid *natural* selection on genes that are transcribed in mature sperm (see also [76]), a phenomenon that has recently been inferred in different taxa by studies reporting that reproductive success differs among sub-populations of phenotypically and genotypically variable sperm ([18–20,77]; but see [78]).

### (b) Sexual selection

Where post-ejaculation gene expression is associated with sperm competitive fertilization ability, we might expect to see within-ejaculate sperm competition [79] and cryptic female choice (female-mediated processes that generate non-random fertilization biases favouring certain sperm over others [80]), both favouring specific sperm cells within a male’s ejaculate. These forms of haploid selection could generate evolutionary conflicts of interest between the male’s haploid and diploid genomes (see below), as individual sperm have a selfish interest in their own chances of fertilization, while the diploid male’s interest lies in maximizing the chance of fertilization by any of his sperm (e.g. [9,11,16]). Despite theoretical interest [79,80], these processes have not received sufficient empirical attention.

### (c) Paternal effects

Other exciting implications of gene expression by mature sperm cells include a change in transcript profiles that are delivered to embryos at fertilization, thus generating ejaculate-mediated paternal effects, i.e. father-offspring transmission of information via the sperm epigenome or the non-sperm fraction of the ejaculate by which a father’s environmental experiences influence his progeny’s phenotype (e.g. development, health or fitness; figure 1; e.g. [22,81,82]). As this review highlights, a growing body of evidence confirms that many RNA transcripts found in mature spermatozoa in mammals and other groups are post-meiotically derived and likely to have important functions during fertilization (reviewed by [10]). Furthermore, such complex RNA populations, which typically include both coding and non-coding RNAs, may also function during intergenerational and transgenerational inheritance, and thus have implications for genotypes and phenotypes of ensuing generations. For example, Fischer *et al*. [6] demonstrated that sperm mRNAs are enriched for protein translation functions in disparate taxa and that such mRNAs can be transmitted to the oocyte, implicating their involvement in translation in early embryos. Furthermore, data emerging from animal models have revealed that non-coding RNAs are sensitive to various environmental factors experienced by males, making them potential sources of epigenetic inheritance [10,83]. However, the capacity for sperm transcript profiles to change *after* ejaculation would provide an entirely new arena of paternal effects induced by the sperm and fertilization environments (e.g. [84,85]). We see this as an exciting area for future research.

### (d) Evolutionary conflicts

The potential for post-ejaculation gene expression to affect both sperm fertilization ability and subsequent offspring development (figure 1) raises possible evolutionary conflicts of interest between males/sperm and females/offspring. For example, genes that affect sperm traits and amplify male competitive fertilization success might not be the same genes that deliver beneficial transcripts to embryos, and in extreme cases, there could be direct trade-offs between expression of genes for sperm function *versus* embryo development or offspring quality. In such cases, we might expect sperm to continue to be selected for expression of genes that benefit fertilization success, owing to strong reproductive competition among males, at a cost to offspring viability and consequently females’ evolutionary fitness (e.g. [86–88]). To our knowledge, such ideas have received almost no empirical attention, although recent findings in external fertilizers provide hints that these processes could indeed occur. As outlined above, Lymbery *et al*. [48] reported that post-ejaculation thermal stress reduces the abundance of heat shock protein transcripts in blue mussel sperm, probably owing to translation of these RNAs to maintain sperm function. However, offspring from heat-treated sperm perform poorly when subsequently reared in high temperatures [89], which might reflect the decreased availability of heat shock transcripts to be delivered to embryos. While these inferences are currently speculative, we suggest that such evolutionary conflicts over sperm gene expression will be a fruitful avenue for future research.

## 7. Practical implications of post-ejaculation sperm gene expression

Several practical considerations for assisted reproduction in humans and other animals have arisen from the discovery that mature spermatozoa contain populations of functioning RNAs [90]. For example, human studies have found that coding and non-coding RNAs in sperm are strongly associated with sperm quality and fertilization success, or differentially expressed in fertile and infertile/sub-fertile men (e.g. [91–94]). Similar findings have been reported in a range of important agricultural or aquacultural animal species [95–97]. Transcriptional profiling may therefore hold promise for the discovery of fertility biomarkers in human clinical studies and animal production (see [10]).

The potential for post-ejaculation sperm gene expression has additional implications for practitioners in assisted reproduction, where sperm are typically exposed to a range of novel conditions associated with collection, storage, preservation and *in vitro* fertilization. As we highlight in this review, there is increasing evidence for changes in sperm transcriptomes after exposure to such procedures (e.g. cryopreservation [41,42,98,99]). These findings suggest there could be previously unappreciated impacts of assisted reproduction techniques on RNAs/proteins with important roles in sperm function and fertility. Therefore, sperm gene expression profiles could be used to determine the optimal procedures for ensuring success of assisted reproduction.

As we outline above, changes in sperm gene expression have potential implications beyond fertilization by generating ejaculate-mediated paternal effects on offspring viability and health [10,22,83]. Therefore, novel environments experienced by sperm during assisted reproduction procedures could have far-reaching consequences if they alter transcripts profiles that are delivered to embryos (for examples of pre-ejaculation environments affecting sperm RNA and offspring phenotypes, see [81,100,101]). Beyond these applied contexts, changes to environmental conditions in wild populations could influence ejaculated sperm gene expression [48] and lead to cross-generational effects on offspring fitness [89,102], generating important implications for the capacity of species to respond to threats such as climate change.

## 8. Conclusions

Our review outlines several lines of evidence that pose potential challenges to the ‘silent sperm’ paradigm—the assumption that gene expression ceases in mature, ejaculated sperm. Studies have unambiguously shown de novo translation of proteins in ejaculated sperm, and a growing body of research indicates that sperm of many species exhibit post-ejaculation phenotypic plasticity of many traits, which could be consistent with post-ejaculation changes in gene expression. Furthermore, assisted reproduction studies increasingly report differential transcript abundance from sperm genes across different post-ejaculation environments, although it is important to note that these studies typically sample sub-fertile or infertile individuals. The key research priority that arises from these findings is to directly determine whether mature, ejaculated, fertile sperm can actively transcribe their nuclear DNA.

We have several recommendations for future studies to test for post-ejaculation transcription, and other key questions identified in this article (see box 3). Studies in both internal and external fertilizers should test for differential expression of sperm genes in response to a broader range of post-ejaculation environments, and critically, should directly test whether such patterns reflect active transcriptional changes (e.g. via labelling nascent RNAs or employing transcription inhibitors). Furthermore, work is needed to determine whether such differential gene expression is correlated with changes in sperm phenotype induced by the post-ejaculation environment, and whether prevention of gene expression (e.g. through RNA interference or transcription/translation inhibitors) impedes phenotypic plasticity. Finally, we require studies that assess the possible evolutionary ramifications of these processes by determining whether changes in gene expression affect fitness in terms of fertilization success and/or offspring viability, and how such effects influence within-ejaculate sperm competition and possible trade-offs between the fitness of sperm, males, females and offspring.

Box 3: **Priorities for future research**

Here, we outline several key questions for future studies that we consider critical to elucidating whether and to what extent sperm gene expression occurs, and how it might affect fertility and evolutionary fitness:

- does de novo transcription occur in ejaculated sperm? If so, how many genes are actively transcribed, and what are their functions?
- do post-ejaculation changes in sperm phenotypes reflect changes in gene expression?
- does the activity of transcription and translation in ejaculated sperm of external fertilizers differ from that of extensively studied internal fertilizers? E.g. is post-ejaculation protein translation in external fertilizers based in the mitochondria?
- if transcription occurs in mature, ejaculated sperm, does it reflect patterns of chromatin compaction in the sperm nucleus?
- does post-ejaculation expression increase the variance in phenotypes and fertilization success among sperm within a male’s ejaculate, and lead to sperm-male evolutionary conflicts or adaptive (anticipatory) effects on embryos (see also priority [vi])?
- can ejaculate-mediated paternal effects on offspring be generated by changes in sperm gene expression induced by the post-ejaculation environment? and
- how do post-ejaculation changes in sperm gene expression affect sperm fertilization success and offspring viability? Are there trade-offs in how expression levels affect these two components of fitness, leading to evolutionary conflicts between males and females/offspring?

## Data Availability

This article has no additional data.
